# Depressed female cynomolgus monkeys (Macaca fascicularis) display a higher second-to-fourth (2D:4D) digit ratio

**DOI:** 10.24272/j.issn.2095-8137.2019.022

**Published:** 2019-05-18

**Authors:** Wei Li, Ling-Yun Luo, Xun Yang, Yong He, Bin Lian, Chao-Hua Qu, Qing-Yuan Wu, Jian-Guo Zhang, Peng Xie

**Affiliations:** 1 Department of Neurology, Army Medical Center of PLA, Chongqing 400042, China; 2 Institute of Neuroscience, Chongqing Medical University, Chongqing 400016, China; 3 Chongqing Key Laboratory of Neurobiology of Chongqing Medical University, Chongqing 400016, China; 4 Department of Neurology, the Third Affiliated Hospital of Sun Yat-Sen Universlty Yuedong Hospital, Guangzhou Guangdong 514700, China

**Keywords:** Finger length, Digit ratio, Major depressive disorder, Cynomolgus monkey

## Abstract

This research aimed to provide evidence of a relationship between digit ratio and depression status in the cynomolgus monkey (*Macaca fascicularis*). In stable cynomolgus monkey social groups, we selected 15 depressed monkeys based on depressive-like behavioral criteria and 16 normal control monkeys. All animals were video recorded for two weeks, with the duration and frequency of the core depressive behaviors and 58 other behaviors in 12 behavioral categories then evaluated via behavioral analysis. Finger lengths from the right and left forelimb hands of both groups were measured by X-ray imaging. Finger length and digit ratio comparisons between the two groups were conducted using Student’s *t*-test. In terms of the duration of each behavior, significant differences emerged in “Huddling” and five other behavioral categories, including Ingestive, Amicable, Parental, Locomotive, and Resting. In addition to the above five behavioral categories, we found that depressed monkeys spent less time in parental and rubbing back and forth behaviors than the control group. Furthermore, the 4th fingers were significantly longer in the left and right hands in the control group relative to the depressed monkeys. The second-to-fourth (2D:4D) digit ratio in the left and right forelimb hands was significantly lower in the control group than that in the depressed group. Our findings revealed significant differences in finger lengths and digit ratios between depressed monkeys and healthy controls, which concords with our view that relatively high fetal testosterone exposure may be a protective factor against developing depressive symptoms (or that low fetal testosterone exposure is a risk factor).

## INTRODUCTION

Major depressive disorder (MDD) is a debilitating psychiatric mood disorder that affects millions of individuals globally (Gelenberg, 2010). Our understanding of the biological basis of MDD is poor, and current treatments are ineffective in a significant proportion of cases. This likely relates to the lack of human and non-human primate research models compared with the dominant rodent models of depression, which possess translational limitations due to limited homologies with humans. Therefore, a more homologous primate model of depression is needed to advance our understanding of the pathophysiological mechanisms underlying depression and to provide a sound basis for conducting pre-clinical therapeutic trials.

Social stress plays a major role in the pathogenesis of depression (Krishnan & Nestler, 2008). In human research, depressive patients, especially women, are more likely to experience depression after prolonged stress (Sherrill et al., 1997). The diagnosis of depression in humans is based on various scales. In line with the DSM-V (Diagnostic and Statistical Manual of Mental Disorders, fifth edition) diagnostic criteria, MDD is characterized by five (or more) of the following symptoms: depressed mood, loss of interest, weight change, sleep disturbance, psychomotor agitation or retardation, loss of energy, feelings of worthlessness, difficulty concentrating, and recurrent thoughts of death. These symptoms must persist for at least two weeks and must include depressed mood and/or loss of interest (Gnanavel & Robert, 2013). In determination of primate depressive behavior, the most reliable method is through behavioral phenotypes. Because of the inability to transfer pressure after being the subject of aggression, depressed female cynomolgus monkeys (*Macaca fascicularis*) can experience long-term social pressure. Shively et al. (2005) indicated that socially-subordinate female cynomolgus monkeys, who are likely weak competitors in social environments due to long-term attack and suppression, can exhibit similar pathogenetic processes as depression in humans. Female cynomolgus monkeys have several behavioral and physiological characteristics in common with human depression. They tend to spend more time alone than their dominant counterparts, exhibit greater vigilance, display slumped or collapsed body posture, show diminished interest in feeding and sex, and subdued communication and reciprocal grooming with others for at least two weeks (Shively et al., 2005). Huddling, which is defined as a slumped body posture with the head at or below the shoulders during the awake state (i.e., when the monkey’s eyes are opened) accompanied by a relative lack in responsiveness to environmental stimuli, is used as a behavioral indicator of depression and is a core posture reflecting depressed mood in monkeys (Felger et al., 2007). Based on these well-established criteria, we successfully constructed a naturally occurring depression model in macaques (Xu et al., 2015). Because of the similarities in complex behavioral and psychological processes between macaques and humans, the development of naturally occurring animal models of disease, whether physical or psychological, is a valuable approach for translational research between human studies and induced primate models, especially for depression (Capitanio, 2017).

The ratio of the index to ring finger length is a commonly used measure. The male ring finger is longer than the index finger, whereas the index finger of the female is basically equal to that of the ring finger. The second-to-fourth (2D:4D) digit ratio was first proposed by Manning et al. (1998) to predict estrogen and sperm number and prenatal sex hormones. Under regulation of the Hox gene, hormone levels during embryonic development, especially the level of androgen, affect finger development (Goodman & Scamble, 2001). The 2D:4D ratio is present in a human embryo by the seventh week of pregnancy (Lutchmaya et al., 2004). Additionally, evidence suggests that in both males and females, the digit ratio can act as an indicator of the level of testosterone the developing fetus was exposed to, making it a useful indirect measure of organizational prenatal hormone exposure. Some research indicates that the 2D:4D ratio may also be an indicator of perinatal androgen action, whereby lower digit ratios predict greater androgen sensitivity (Smedley et al., 2014). Thus, given that depression is a strongly sexually dimorphic trait, it is reasonable to expect that the 2D:4D ratio may be related to depression (Evardone & Alexander, 2009). Furthermore, previous investigations have confirmed that the 2D:4D ratio is associated with human behavior, such as aggression, cooperation, left-handedness, and human disease, including breast cancer, dyslexia, infertility, myocardial infarction, and autism (Ronalds et al., 2002). For instance, Bailey & Hurd (2005) reported that the 2D:4D ratio is related to individual aggressiveness, confidence, and competitive ability. Furthermore, other developmental disorders, emotional behaviors, and negative and affective symptoms in schizophrenia are reported to be related to the 2D:4D ratio (Carre et al., 2015). Previous studies have also shown that individuals with a higher 2D:4D ratio are more likely to suffer from depression. For instance, Smedley et al. (2014) found that a higher digit ratio is correlated with higher depression scores in females, but not males. To date, however, results have been inconsistent. For example, using a large sample comprised of 298 college students (149 males and 149 females), Bailey & Hurd (2005) found that more feminine ratios were associated with higher depression in men, but found no correlation between the 2D:4D ratio and depression in women. Bailey & Hurd (2005) produced the unusual finding of no sex differences in depression, although digit ratios did differ in the expected direction. As such, it remains unclear to what degree depression and the 2D:4D ratio, both characterized by marked sex differences, are related (Smedley et al., 2014).

Females are more susceptible to depression in social groups and depression is approximately twice more common among women than men (Trivers et al., 2006). Therefore, we chose female cynomolgus monkeys as our research targets. Based on our previous method (Xu et al., 2015), a total of 15 depressed female monkeys were selected across 52 enclosures, with 16 healthy subjects selected as controls from the original population (*n*=6 012). A strict radiographic procedure was used to measure index and ring finger lengths in both forelimb hands. This investigation aimed to provide evidence of a relationship between the digit ratio and depression status in the cynomolgus monkey.

## MATERIALS AND METHODS

### Ethics statement

Behavioral data acquisition was observational under normal circumstances and did not involve physical manipulation of the subjects or changes to their environment or diet. Animal care and housing procedures followed Chinese regulatory requirements and the Association for Assessment and Accreditation of Laboratory Animal Care International. In brief, complete animal husbandry and veterinary care were provided daily. Animals were fed a nutritious standardized diet, supplemented daily with fresh fruits and vegetables. Animals had unrestricted access to potable water and their enclosures were cleaned each day. Animals were observed daily by trained care-takers. Any observed abnormality, disease, or injury was reported to the veterinary staff for diagnosis and treatment; this veterinary support was documented in both hard copy and electronic formats. In addition, this study was performed in strict accordance with the recommendations in the Guide for the Care and Use of Laboratory Animals of the Institute of Neuroscience of Chongqing Medical University (Approval No.: 20100031). Prior to implementation, the experimental protocol was approved by the Committee on the Ethics of Animal Experiments at Chongqing Medical University and was in accordance with state regulations.

### Observation site

The *M. fascicularis* Feeding Base of Zhongke Experimental Animal Co., Ltd. is in Suzhou, China, at E31º07′03″ to E31º07′06″, N120º19′08″ to N120º19′15″. The company imported the *M. fascicularis* subjects from Guangdong Province, China and from Vietnam in 1990, from which they established a domestication and breeding base for these monkeys.

### Subjects

**Figure 1:**
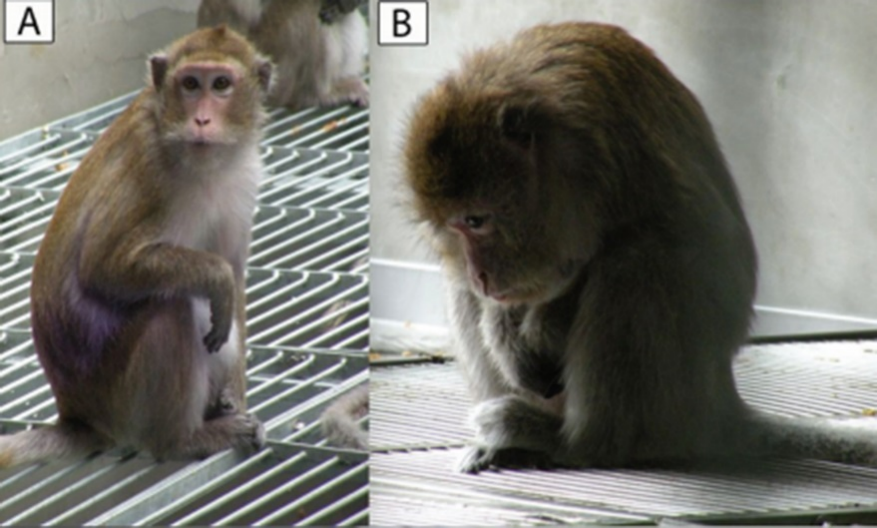
**Core depressive behavior (**“**Huddling**”**) in a cynomolgus monkey**

We scanned a total population of 6 012 adult female cynomolgus monkeys across all 52 enclosures. Depressive behavior was identified using the operational definition according to Shively’s criteria: slumped or collapsed body posture (Figure 1), diminished interest in feeding and sex, and diminished communication and reciprocal grooming with others (Xu et al., 2015). Sixteen healthy adult female *M. fascicularis* subjects (aged 9–13 years) were randomly selected from the pool of 6 012 monkeys. A total of 15 depressed female monkeys (aged 10–12 years) were selected from the 52 enclosures based on the above-mentioned depression phenotypes lasting for at least two weeks. All subjects were reared in socially-stable colonies with negligible rates of conflict (Willard & Shively, 2012). Staff veterinarians ruled out disease in the subjects. Each colony was housed in an indoor free enclosure measuring 8.0 m×3.0 m×3.0 m (L×W×H) with continuous daylight exposure. Every colony was composed of two males, 16–22 adult females, and their offspring of less than six months of age. To reflect wild populations, the male:female ratio was maintained at 1:(7–10).

A: Normal cynomolgus monkey; B: Depressed cynomolgus monkey exhibiting “Huddling” behavior.

Behavioral recording methods and scored behavioral items are described in our previous work (Xu et al., 2012). The duration and frequency of the core depressive behavior “Huddling” (Figure 1) and 58 other behaviors in 12 categories (Ingestive, Thermoregulatory, Rutting and estrous, Mating, Resting, Parental, Amicable, Conflict, Vigilance, Communication, Locomotive, Miscellaneous behaviors) were video recorded by three well-trained observers blind to the behavioral definition using NOLDUS Observer XT software (v10.0, Noldus Information Technology, Leesburg, PA, USA) during two consecutive weeks with four phases per day (A1 0900–0930 h; A2 0930–1000 h; P1 1500–1530 h; P2 1530–1600 h). Behavioral data were coded as duration (in seconds) and frequency (in count) for each discreet behavioral item per 30 min observational phase and presented as means±*SD*.

### Finger length measurement with radiography

**Figure 2:**
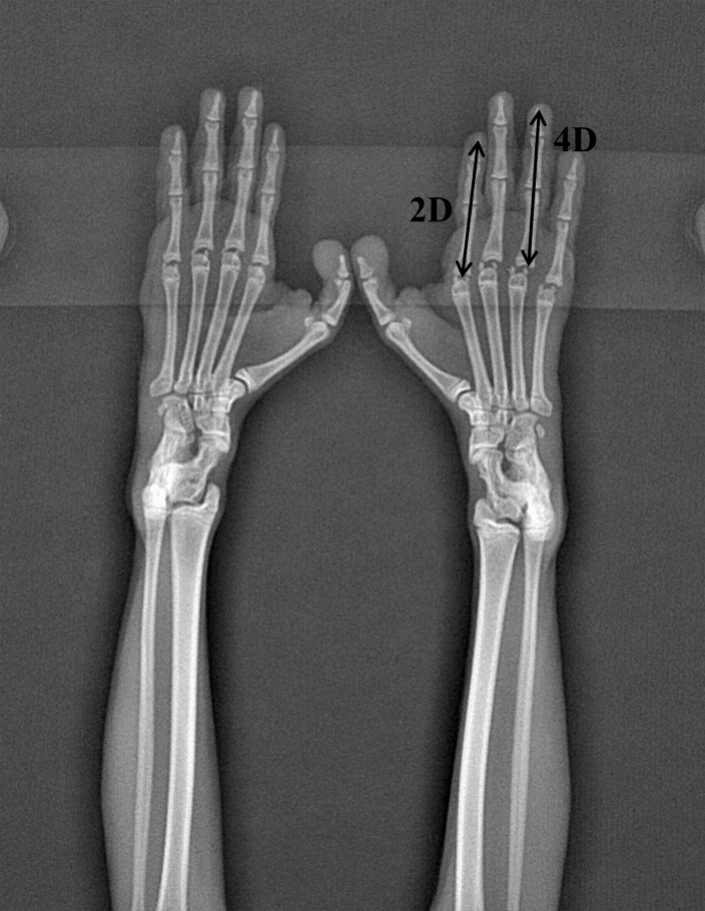
Forelimb hand X-ray image

We measured index and ring finger lengths from the right and left forelimb hands in both groups after the last day of behavioral data acquisition using a digital radiography unit with a flat-panel digital detector (PLX8200, Perlove, Nanjing, China) (Kalichman et al., 2017). The digital detector was exposed to X-ray at 60 kVp, with an approximate detector-to-tube distance of 1 m. Exposure times were no greater than 0.1 s, resulting in 4.0 mA exposure. Three qualified staff performed this process in cooperation: one undertook anesthesia and hand-position adjustment, one operated the machine to acquire finger length, and one recorded the data and was blind to the experiment. Ketamine (10 mL/kg) anesthetic was administered intramuscularly (I.M.) in the distal hind limb at 0900 h (Nelson & Voracek, 2010). Approximately 10 min after ketamine injection, the finger length ratio was measured at an accuracy of 0.01 mm using X-ray imaging (Choi et al., 2011) (Figure 2).

2D: Index finger; 4D: Ring finger.

### Statistical methods

To assess the behavioral differences between depressed subjects and healthy controls, Student’s *t*-test was performed if the data were normally distributed; otherwise, the Mann-Whitney *U* test was applied. As there were 58 behavioral items in total, Bonferroni correction was used to reduce type I errors for multiple comparisons. Primate fingers are termed 1 through 5 from the thumb to the smallest digit, respectively. In this investigation, the 2D:4D digit ratio was used (Kyriakidis et al., 2010). To minimize error, all finger length data were presented as means±*SD*. Finger length and digit ratio comparisons between the two groups were conducted using Student’s *t*-test. *P* values of less than 0.05 were deemed significant for all data. Data management and statistical analysis were performed using SPSS 21.0.

## RESULTS

### Differences in behavioral phenotype between depressed and control monkeys

In terms of the duration of behavior, depressed monkeys spent more time “Huddling” (*P*<0.001), in which the target displayed a fetal-like, self-enclosed posture with a lowered head during the awake state (i.e., when the monkey’s eyes were opened). The increased duration of “Huddling” indicated that the depressed subjects recursively stayed in the depressed mood condition. For Ingestive behavior, the control subjects preferred to “feed while sitting” (*P*<0.05), whereas the depressed subjects displayed more vigilance and preferred “feeding while hanging” to avoid potential threats and attacks. They also spent more time “licking food residue off the cage” (*P*<0.05) due to the pressure of other monkeys during normal food intake. In accordance with the depression criteria, depressed monkeys also spent less time drinking. The impact of pressure on both groups was further reflected in resting behavior, with the depressed group more reluctant to “hang on iron chain rest” (*P*<0.001) compared to the control group, but more willing to choose a remote area for resting, namely, “hanging on skylight rest” (*P*<0.001). Regarding locomotive behavior, depressed individuals exhibited less vitality in walking and standing and spent relatively less time performing “quadrupedal walking on floor” (*P*<0.001), “walking on iron chain” (*P*<0.001), and “standing” (*P*<0.05). Indicative of a friendly relationship among others, the control group received more amicable grooming (i.e., “being groomed” (*P*<0.001)) and groomed others more often (i.e., “mutual grooming” (*P*<0.001)) than exhibited by the depressed group, suggesting reduced interaction in depression. Furthermore, there was a reduction in the duration of “nursing” (*P*<0.05) parental behavior. For mating behavior, which is a sign of sexual interest, the duration of “copulation” (*P*<0.05) was higher in the control monkeys in comparison to the depressed females, with a significant difference in the frequency of mating behavior also observed (Table 2). In the end, except the frequency of behaviors matched with the duration of behaviors, a lower frequency of miscellaneous behavior (i.e., “rub hand back and forth” (*P*<0.001)) existed in depressed individuals, suggesting that depressed monkeys may be less imposing and lacking in confidence.

**Table 1:** Duration of behaviors observed in depressed and control subjects

Behavior		Duration			
Behavioral category	Behavior	Depressed group	Control subjects	*P* value	Adjusted *P* value
Core behavior	Huddling***	365.44±514.14	159.00±355.08	*P*<0.001	*P*<0.001
Ingestive behavior	Drinking***	2.51±13.09	5.92±21.59	*P*<0.001	*P*<0.001
	Feeding while hanging*	6.16±33.19	3.56±24.14	*P*=0.0004	*P*=0.0243
	Lick food residue off cage*	1.29±13.78	0.44±3.24	*P*=0.0007	*P*=0.0419
	Feeding while sitting***	29.73±89.06	63.29±171.94	*P*<0.001	*P*<0.001
Amicable behavior	Mutual grooming***	51.96±153.61	114.13±226.53	*P*<0.001	*P*<0.001
	Being groomed***	51.75±120.17	97.94±198.63	*P*<0.001	*P*<0.001
Parental behavior	Nursing*	7.33±36.69	11.75±57.42	*P*=0.0006	*P*=0.0334
Locomotive behavior	Quadrupedal walking on floor***	62.82±58.99	82.03±75.68	*P*<0.001	*P*<0.001
	Walking along iron chain***	0.13±1.26	0.85±5.62	*P*<0.001	*P*<0.001
	Standing*	9.75±25.35	13.94±29.95	*P*=0.0006	*P*=0.0378
Resting behavior	Hanging on iron chain rest***	5.85±71.70	45.35±176.59	*P*<0.001	*P*<0.001
	Hanging on skylight rest***	23.18±143.93	8.88±81.59	*P*<0.001	*P*<0.001

Data are means±*SD*. Bonferroni correction was used to reduce type I errors for multiple comparisons. Mann-Whitney *U* test, *: *P*<0.05; **: *P*<0.01; ***: *P*<0.001*.*

**Table 2:** Frequency of behaviors observed in depressed and control subjects

Behavior		Frequency			
Behavioral category	Behavior	Depressed group	Control subjects	*P* value	Adjusted *P* value
Core behavior	Huddling***	1.53±2.17	0.51±1.25	*P*<0.001	*P*<0.001
Ingestive behavior	Feeding while hanging***	0.11±0.63	0.05±0.28	*P*<0.001	*P*<0.001
	Feeding while sitting**	0.85±2.15	1.11±2.47	*P*<0.001	*P*<0.01
	Drinking***	0.20±0.86	0.51±1.59	*P*<0.001	*P*<0.001
Amicable behavior	Mutual grooming***	0.74±1.83	1.33±2.40	*P*<0.001	*P*<0.001
Mating behavior	Copulation*	0.08±0.35	0.12±0.53	*P*=0.0007	*P*=0.0418
Parental behavior	Nursing*	0.28±1.01	0.40±1.44	*P*=0.0002	*P*=0.0129
Resting behavior	Hanging on iron chain rest***	0.02±0.22	0.23±0.80	*P*<0.001	*P*<0.001
	Hanging on skylight rest***	0.29±1.63	0.10±0.62	*P*<0.001	*P*<0.001
Locomotive behavior	Walking along iron chain***	0.03±0.21	0.10±0.53	*P*<0.001	*P*<0.001
Miscellaneous behavior	Rub hand back and forth***	0.02±0.15	0.06±0.43	*P*<0.001	*P*<0.001

Data are means±*SD*. Bonferroni correction was used to reduce type I errors for multiple comparisons. Mann-Whitney *U* test, *: *P*<0.05; **: *P*<0.01; ***: *P*<0.001*.*

### Finger length data

Finger length was measured in the 16 control and 15 depressed animals (Table 3). For both the left and right hand, the ring finger was significantly longer in the control group than that in the depressed group.

**Table 3:** Digit length in right and left forelimbs of depressed and control subjects (cm)

Left/Right	Digit^†^	Control (*n*=16)		Depressed (*n*=15)	
		Mean	*SD*	Mean	*SD*
Left	2	29.76	2.46	29.28	2.62
	4*	37.75	2.60	35.45	3.17
Right	2	30.25	2.67	29.04	2.78
	4*	37.14	2.64	35.09	3.24

^†^: Fingers are numbered 1 to 5 from thumb to smallest digit. Data are means±*SD*. Student’s *t*-test, *: *P*<0.05.

### Digit ratio comparison

The digit ratio was significantly lower in the control group than in the depressed monkeys ( Table 4), including the 2D:4D ratio in the right and left forelimb hands of depressed and control subjects.

**Table 4:** 2D:4D ratio in the right and left forelimb hands of depressed and control subjects

Left/ Right	Digit Ratio	Control			Depressed			*P* value
		*n*	Mean	*SD*	*n*	Mean	*SD*	
Left	2D:4D	16	0.79	0.04	15	0.83	0.03	0.002**
Right	2D:4D	16	0.81	0.03	15	0.84	0.05	0.0372*

Data are means±*SD*. Student’s *t*-test, *: *P*<0.05; **: *P*<0.01; ***: *P*<0.001*.*

## DISCUSSION

With respect to primate finger length investigations, the 2D:4D ratio is strongly related to social behavior and physical aggression. Nelson & Shultz (2010) reported that a low 2D:4D ratio is associated with more competitive social systems, which is in accordance with our previous observation (Zhou et al., 2014) that depressed monkeys face greater competition for social resources–including feeding opportunities, comfortable resting places, and mating opportunities–and display significant deficits in social interactions. Thus, the cynomolgus monkey population is a suitable choice to study the relationship between the 2D:4D ratio and depression.

In the present study, we employed a reliable naturally occurring primate depression model to identify depressed cynomolgus monkeys. Our data disclosed that the ring fingers in both the left and right forelimb hands were longer in healthy female monkeys than in depressed female monkeys. In terms of the finger length data, we found that the digit ratios were significantly higher in depressed monkeys, including the 2D:4D ratios in the left and right forelimb hands. Depression can occur due to long-term social pressures, especially in females (Sherrill et al., 1997). In a competitive environment, the dorsal anterior cingulate cortex (dACC) region in the brain controls support, emotion regulation, conflict monitoring, and behavioral inhibition (Dedovicl et al., 2016). Gorka et al. (2015) revealed a significant positive correlation between the 2D:4D ratio and gray matter volume of the dACC in women but not in men. Interestingly, maturation of the dACC influences the development of MDD (Ho et al., 2017). The critical hippocampal brain area, which is strongly associated with the pathogenesis of depression, is also related to the 2D:4D ratio (Kallai et al., 2005). These studies provide a possible intrinsic link in the brain tissue between 2D:4D ratio and depression.

Previous investigations have shown that the digit ratio persists in a stable range during embryogenesis and increases in accordance with personal growth (Goodman & Scamble, 2001; Herault et al., 1997). Interestingly, Williams et al. (2000) used the 2D:4D ratio to reflect the degree of prenatal androgen exposure in humans. However, a growing body of evidence indicates that the 2D:4D ratio is unrelated to adult sex hormone (e.g., estrogen and androgen) concentrations (Muller et al., 2011). The 2D:4D ratio appears to be relatively stable, although it does increase somewhat throughout childhood (Trivers et al., 2006). Thus, there is a general consensus that the 2D:4D ratio is a relatively stable biomarker for the balance between fetal testosterone (FT) and fetal estrogen (FE), with low FT and high FE linked to high 2D:4D (Manning et al., 2014). Based on these findings, we hypothesize that the 2D and 4D finger length ratios are primarily determined by prenatal sex hormone exposure, and that the effects of this prenatal hormone on the 2D:4D ratio are not presented as estrogen or androgen concentrations differences in a later period. However, the prenatal sex hormon affect the subject's neural development and biochemistry (Honekopp et al., 2007). Those with longer finger lengths tend to possess poor aggression tendencies and emotion regulation function. This is also consistent with our findings. In a competitive environment, high 2D:4D female individuals exhibited a high correlation with depression. Thus, our findings provide a novel way in which to select depressed monkeys according to comparison of the 2D:4D ratio. Future work should examine the relationship between the 2D:4D ratio and the severity of depression in larger samples that report a wider range of depression symptoms. Measurement of the 2D:4D ratio may provide a predictive tool for the diagnosis of depression and strong support for indications of depression risk to proceed early intervention.

## CONCLUSIONS

Most previous primate digit ratio studies have been examined in regard to social behaviors and rank. However, few have investigated the relationship between digit ratios and depression in primates. This is the first study to reveal significant differences in finger lengths and digit ratios between depressed monkeys and healthy controls. We discovered that depressed monkeys presented with shorter 4th fingers and a higher 2D:4D ratio in both forelimbs. These metrics show promise as gross biological indicators to facilitate screening for depressed monkeys in large population-based studies. However, whether this conclusion can be applied to screen for human depression requires further investigation.
